# Comparative biocompatibility of hydrophilic vs. hydrophobic intraocular lenses following phacoemulsification in patients with uveitis

**DOI:** 10.1371/journal.pone.0331586

**Published:** 2025-09-08

**Authors:** Atitaya Apivatthakakul, Apichat Tantraworasin, Paradee Kunavisarut, Kessara Pathanapitoon

**Affiliations:** 1 Department of Ophthalmology, Faculty of Medicine, Chiang Mai University, Chiang Mai, Thailand; 2 Department of Surgery, Faculty of Medicine, Chiang Mai University, Chiang Mai, Thailand; 3 Clinical Surgical Research Center, Faculty of Medicine, Mai University, Chiang Mai, Thailand Chiang; University of Warmia, POLAND

## Abstract

**Purpose:**

To evaluate and compare the biocompatibility of hydrophilic and hydrophobic intraocular lenses (IOLs) in patients with uveitis undergoing phacoemulsification, with particular focus on posterior capsule opacification (PCO), postoperative inflammation, and visual outcomes.

**Methods:**

Patients with uveitis who underwent phacoemulsification with IOL implantation between 2015 and 2023 were retrospectively reviewed. Propensity score matching (1:1) was performed to account for clinical and demographic variables, yielding 132 eyes (66 per group) for analysis. Primary outcomes included the incidence of PCO and the need for neodymium-doped yttrium aluminum garnet (Nd:YAG) laser capsulotomy. Secondary outcomes were the rate of uveitis reactivation and visual improvement. Cox regression models were used to estimate adjusted hazard ratios (HRs) with 95% confidence intervals (CIs).

**Results:**

Hydrophobic IOLs were significantly associated with a reduced risk of PCO (adjusted HR = 0.35; 95% CI, 0.18–0.68; p < 0.05) and Nd:YAG capsulotomy (adjusted HR = 0.23; 95% CI, 0.09–0.56; p < 0.05) compared to hydrophilic IOLs. No significant difference was found in the rate of uveitis reactivation between groups (adjusted HR = 0.83; 95% CI, 0.37–1.88; p = 0.643).

**Conclusion:**

Hydrophobic intraocular lenses show better capsular biocompatibility in uveitic eyes by significantly reducing the incidence of PCO and need for Nd:YAG capsulotomy, without increasing postoperative inflammation. These findings support the preferential use of hydrophobic acrylic IOLs in cataract surgery for patients with uveitis.

## Introduction

Uveitis, a group of intraocular inflammatory disorders, is a major cause of preventable visual impairment, responsible for approximately 10–15% of blindness in developed countries [1,2]. One of the most frequent ocular complications in uveitic patients is cataract formation, which may result from chronic intraocular inflammation or the long-term use of corticosteroids [3,4]. Phacoemulsification with intraocular lens (IOL) implantation is the standard of care for restoring visual function; however, selecting the appropriate IOL material is critical, especially in eyes with heightened inflammatory potential [5,6]. Two commonly used materials for IOLs—hydrophilic and hydrophobic acrylic—differ in their postoperative performance and tissue interaction, particularly in high-risk eyes.

Posterior capsule opacification (PCO), the most common long-term complication following cataract surgery, occurs due to migration and proliferation of residual lens epithelial cells on the posterior capsule, leading to visual axis opacification and the need for neodymium-doped yttrium aluminum garnet (Nd:YAG) laser capsulotomy [7,8]. The rate of PCO is influenced by IOL material, edge design, and surgical factors [7,9–11]. Hydrophilic IOLs, while flexible and easy to insert, have been associated with increased PCO formation in both general and uveitic populations [12,13]. Conversely, hydrophobic IOLs demonstrate a lower rate of epithelial cell adhesion and proliferation, which may account for their reduced PCO rates [8,14].

In addition to PCO, postoperative inflammation is a significant concern in uveitic patients. These eyes are predisposed to exaggerated inflammatory responses, including anterior chamber cells and flare, synechiae, and macular edema [15]. Several studies have reported that hydrophobic IOLs are better tolerated in uveitic eyes, with lower anterior segment inflammation and fewer postoperative complications [14,16]. Nevertheless, other reports suggest that with adequate perioperative immunosuppression, hydrophilic lenses can perform comparably in terms of safety and inflammation control [13,17].

Biocompatibility refers to the ability of an implanted IOL material to maintain its optical function in vivo without eliciting significant adverse reactions such as anaphylaxis, inflammation, immune rejection, or thrombosis [18]. Capsular biocompatibility, in particular, is influenced by the direct interaction between the IOL and residual lens epithelial cells (LECs). This contact can promote cellular adhesion, potentially leading to anterior capsule opacification (ACO) and PCO [19].

Given these considerations, this study aims to compare the biocompatibility of hydrophilic versus hydrophobic acrylic IOLs in patients with uveitis undergoing phacoemulsification. The primary outcome is the rate of posterior capsule opacification, and the secondary outcome is postoperative inflammation, final visual acuity. This comparison seeks to inform evidence-based surgical decisions for optimizing visual outcomes and minimizing complications in uveitic eyes.

Cataract surgery with primary IOL implantation has shown good results in last decades. However, questions remain regarding optimal IOL material in the setting of uveitis. The purpose of this article was to compare the development of PCO, uveal biocompatibility and visual outcomes after implanting IOL between hydrophilic and hydrophobic intraocular lens in patients with uveitis.

## Methods

### Patients and data collection

This retrospective cohort study included patients with uveitis who underwent uneventful phacoemulsification with IOL implantation between January 2015 and December 2023 at a tertiary care referral center. Data were accessed for research purposes from February 1^st^, 2021 to December 15^th^, 2024. In cases where both eyes had undergone cataract surgery, one eye was randomly selected for analysis using a computer-generated sequence to avoid inter-eye correlation. Patients were excluded if they had irreversible ocular conditions causing visual impairment at baseline (e.g., macular scarring, macular ischemia, corneal opacification, optic disc atrophy, or optic neuropathy), baseline total blindness, were under 18 years of age, had ocular conditions mimicking uveitis, experienced intraoperative complications, active inflammation or had less than three months of follow-up. This study was reviewed and approved by the Research Ethics Committee, Faculty of Medicine, Chiang Mai University, Chiang Mai, Thailand (No: OPT-2564–07869), with a waiver for written informed consent due to its retrospective nature. This study was conducted in accordance to the Declaration of Helsinki and the patient data was maintained with confidentiality.

Data were collected from electronic medical records, including patient age at surgery, sex, laterality of ocular involvement, presence of preoperative posterior synechiae, intraocular pressure, and the anatomical site of maximum inflammation (anterior, intermediate, posterior, or panuveitis). Additional data included underlying etiology, preoperative, intraoperative and postoperative treatments, and postoperative clinical outcomes. Corrected distance visual (CDVA) acuity was assessed using the Snellen chart and recorded at the initial presentation, preoperative evaluation, and final follow-up visit; all values were converted to logarithm of the minimum angle of resolution (logMAR) units for analysis. Postoperative complications including posterior capsule opacification and the need for Nd:YAG capsulotomy, active inflammation despite under treatment were documented. Since the presence of PCO does not always necessitate Nd:YAG capsulotomy, particularly in the absence of significant visual impairment. We categorized the primary outcomes into two groups: presence of PCO and PCO requiring Nd:YAG treatment. Quiescence was defined as the presence of ≤ 1 + cells (5–10 cells per high-power field) in the anterior chamber and no active vitritis, as observed using slit lamp biomicroscope with a narrow-slit beam. Recurrent uveitis was defined as episodes of inflammation following surgery separated by at least three months of inactivity, in accordance with SUN criteria [20].

### Surgery

All patients included in the study had quiescent intraocular inflammation for at least three months prior to cataract surgery. Phacoemulsification with IOL implantation was performed using a standardized technique by one of two experienced surgeons (KP or AA). Surgeon 1 performed 91 procedures, of which 52 eyes received the hydrophilic IOL, while surgeon 2 performed 83 procedures, with 47 eyes receiving the same model.

The hydrophilic IOL used was the Bi-Flex AB (Medicontur Medical Engineering Ltd., Budapest, Hungary), a single-piece, foldable, aspheric hydrophilic acrylic lens with a 25% water content and a 360° square edge design. The hydrophobic IOL used was the Sensar AR40e (Johnson & Johnson Vision, Santa Ana, CA, USA), a three-piece, foldable, hydrophobic acrylic lens with OptiEdge™ design, characterized by a posterior square edge, and PMMA monofilament haptics.

Topical anesthesia with 0.5% tetracaine hydrochloride was administered, followed by a subconjunctival injection of 2% lidocaine hydrochloride. Corneoscleral incisions measuring between 2.6 mm and 3.2 mm were used, depending on the period during which the surgery was performed and a 5-mm continuous curvilinear capsulorhexis was created under sodium hyaluronate viscoelastic protection. Standard hydrodissection and hydrodelineation were performed using balanced salt solution (BSS). Phacoemulsification was carried out within the capsular bag using BSS containing 0.5 mg of adrenaline per 500 mL as the intraocular irrigating solution. In all cases, an IOL was implanted in the capsular bag, selected according to the surgeon’s preference.

Postoperatively, all patients were treated with topical prednisolone acetate 1% for at least one month, with tapering based on the clinical course. Postoperative systemic corticosteroids were administered at the discretion of the operating surgeon.

### Statistical analysis

All statistical analyses were conducted using Stata Statistical Software version 16.1 (StataCorp, College Station, TX, USA). Continuous variables were summarized as mean ± standard deviation (SD) if normally distributed, or as median with interquartile range (IQR) for skewed data. Categorical variables were presented as frequencies and percentages. Group comparisons for continuous variables were performed using the independent Student’s t-test for normally distributed data and the Mann–Whitney U test for non-normally distributed data. Categorical variables were compared using the Chi-square test or Fisher’s exact test, as appropriate. A two-sided p-value < 0.05 was considered statistically significant.

To adjust for baseline differences and reduce confounding, 1:1 propensity score matching (PSM) was performed using a nearest-neighbor algorithm without replacement. Covariates included in the propensity score model were age at surgery, sex, anatomical site of uveitis, preoperative use of corticosteroids and immunomodulatory therapy, presence of preoperative posterior synechiae, and operating surgeon. The balance between matched groups was assessed using standardized mean differences (SMDs), with an SMD < 0.1 considered indicative of adequate balance. Matched cohorts were then used for all subsequent outcome analyses to ensure comparability between the hydrophilic and hydrophobic intraocular lens groups.

## Results

### Characteristics of the enrolled patients

A total of 174 eyes from patients with uveitis who underwent phacoemulsification with IOL implantation were included in the study prior to PSM, comprising 99 eyes in the hydrophilic IOL group and 75 eyes in the hydrophobic IOL group. After 1:1 PSM, 132 eyes (66 in each group) remained for the matched analysis.

Propensity score matching was performed to minimize baseline differences between groups and control for potential confounding variables. The covariates selected for matching included patient age at the time of surgery, sex, anatomical site of uveitis, preoperative use of corticosteroids and immunomodulatory drugs, presence of preoperative posterior synechiae, and operating surgeon. These variables were chosen based on their known clinical relevance and potential influence on IOL selection.

Table 1 summarizes the baseline characteristics before and after PSM. Prior to matching, most covariates were relatively balanced between groups, although standardized mean differences (SMDs) for variables such as iris retractor use (SMD = 0.267) and postoperative steroid use (SMD = 0.275) indicated some imbalance. Following matching, the groups were well balanced, with all SMDs below 0.25 and most below 0.1, indicating a high degree of covariate balance.

**Table 1:** Baseline characteristics of patients receiving hydrophilic versus hydrophobic intraocular lenses before and after propensity score matching (PSM).

Characteristics	Pre propensity score matching					Post propensity score matching				
	Total (N = 174)	Hydrophillic IOL (N = 99)	Hydrophobic IOL (N = 75)	P-value	Standardized mean difference	Total (N = 132)	Hydrophillic IOL (N = 66)	Hydrophobic IOL (N = 66)	P-value	Standardized mean difference
Covariates selected in PSM										
Age at surgery (year), Mean±SD	54.15 ± 16.73	53.9 ± 16.65	54.4 ± 16.95	0.57	0.026	54.02 ± 15.98	53.73 ± 15.49	54.31 ± 16.56	0.83	0.037
Male, n (%)	69 (39.66%)	41 (41.41%)	28 (37.33%)	0.586	0.083	49 (37.12%)	22 (33.33%)	27 (40.91%)	0.368	0.156
Uveitis site, n (%)				0.03	0.247				0.12	0.010
Anterior uveitis	94 (54.02%)	58 (58.59%)	36 (48.0%)			71 (53.79%)	35 (53.03%)	36 (54.55%)		
Intermediate uveitis	1 (0.57%)	0 (0%)	1 (1.33%)			1 (0.76%)	0 (0%)	1 (1.52%)		
Posterior uveitis	9 (5.17%)	8 (8.08%)	1 (1.33%)			8 (6.06%)	7 (10.61%)	1 (1.52%)		
Panuveitis	70 (40.23%)	33 (33.33%)	37 (49.33%)			52 (39.39%)	24 (36.36%)	28 (42.42%)		
Pre operative posterior synechiae, n (%)	144 (82.76%)	82 (82.83%)	62 (82.67%)	0.98	0.004	108 (81.82%)	53 (80.30%)	55 (83.33%)	0.65	0.078
Pre operative steroid used, n (%)				0.12	0.269				0.18	0.083
No steroid used	8 (4.60%)	4 (4.04%)	4 (5.33%)			3 (2.27%)	2 (3.03%)	1 (1.52%)		
Topical steroid used	41 (23.56%)	19 (19.19%)	22 (29.33%)			29 (21.97%)	13 (19.70%)	16 (24.24%)		
Systemic steroid used	2 (1.15%)	0 (0.00%)	2 (2.67%)			2 (1.52%)	0 (0.00%)	2 (3.03%)		
Topical with systemic steroid used	123 (70.69%)	76 (76.77%)	47 (62.67%)			98 (74.24%)	51 (77.27%)	47 (71.21%)		
Preoperative immunomodulatory drug used, n (%)	103 (59.20%)	60 (60.61%)	43 (57.33%)	0.66	0.066	79 (59.85%)	39 (59.09%)	40 (60.61%)	0.66	0.031
Surgeon, n (%)				0.65	0.100				0.73	0.060
1	91 (52.30%)	52 (52.53%)	39 (52.0%)			72 (54.55%)	37 (56.06%)	35 (53.03%)		
2	83 (47.70%)	47 (47.47%)	36 (48.0%)			60 (45.45%)	29 (43.94%)	31 (46.97%)		
Other Covariates										
Initial CDVA (median (IQR))	0.8 (0.6-1.6)	1.0 (0.5-1.6)	0.8 (0.6-1.0)	0.79	0.125	0.9 (0.6-1.5)	1.0 (0.5-1.6)	0.8 (0.6-1.0)	0.87	0.095
Pre operative CDVA (median (IQR))	1.0 (0.8-1.8)	1.0 (0.8-1.8)	1.0 (0.8-1.8)	0.31	0.111	1.0 (0.8-1.8)	1.0 (0.8-1.8)	1..0 (1.0-1.8)	0.32	0.100
Bilateral	147 (84.48%)	84 (84.85%)	63 (84.0%)	0.88	0.023	112 (84.85%)	58 (87.88%)	54 (81.82%)	0.33	0.168
Initial intraocular pressure rising, n (%)	56 (32.18%)	33 (33.33%)	23 (30.67%)	0.71	0.057	49 (35.61%)	25 (37.88%)	22 (33.33%)	0.71	0.057
Iris retractor use, n (%)	78 (44.83%)	50 (50.51%)	28 (37.33%)	0.06	0.267	58 (43.94%)	33 (50.00%)	25 (37.88%)	0.16	0.244
Intraoperative steroid used, n (%)	145 (83.33%)	84 (84.85%)	61 (81.33%)	0.54	0.093	110 (83.33%)	55 (83.33%)	55 (83.33%)	1.00	0.00
Post operative steroid used, n (%)				0.1	0.275				0.10	0.291
Topical steroid used	28	12	16			21 (15.91%)	7 (10.61%)	14 (21.21%)		
Topical with systemic steroid used	146	87	59			111 (84.09%)	59 (89.39%)	52 (78.79%)		
Follow up time after surgery (months), median (IQR)	33.6 (18.1-58.13)	32.8 (13.6-56.33)	34.4 (23-63.67)	0.09	0.32	33.6 (18.1-58.13)	32.08 (15.0 −47.97)	34.97 (23.17-63.67)	0.07	0.33

Abbreviations: CDVA: Corrected distance visual acuity; IOL: Intraocular lens; IQR: Interquartile range; PSM: Propensity score matching; SD: Standard deviation

The mean age at surgery remained similar between the hydrophilic and hydrophobic groups both before and after matching. Sex distribution was also comparable post-matching, with males comprising 33.33% in the hydrophilic group and 40.91% in the hydrophobic group (p = 0.368).

Before PMS, the distribution of the anatomical site of uveitis differed slightly between groups, with anterior uveitis being the most common in both cohorts. Specifically, anterior uveitis was observed in 58.6% of the hydrophilic group and 48.0% of the hydrophobic group. Panuveitis was present in 33.3% and 49.3% of eyes in the hydrophilic and hydrophobic groups, respectively. Posterior uveitis was more frequent in the hydrophilic group (8.1%) compared to 1.3% in the hydrophobic group, while intermediate uveitis was rare, reported in only one patient (1.3%) in the hydrophobic group. The overall distribution of uveitis sites differed significantly between groups prior to matching (*p* = 0.03; standardized mean difference [SMD] = 0.247).

After PSM, the groups were well balanced regarding uveitis site. Anterior uveitis remained the predominant diagnosis in both groups (53.0% in the hydrophilic group vs. 54.6% in the hydrophobic group), followed by panuveitis (36.4% vs. 42.4%). Posterior and intermediate uveitis continued to be less frequent, with posterior uveitis occurring in 10.6% of the hydrophilic group and 1.5% of the hydrophobic group, and intermediate uveitis reported in only one patient in the hydrophobic group (1.5%). The difference in anatomical distribution was no longer statistically significant after matching (*p* = 0.12), and the SMD improved to 0.010, indicating excellent balance between the matched groups.

There were no significant differences between the groups in the rate of preoperative posterior synechiae (80.3% vs. 83.3%, p = 0.65), preoperative use of systemic or topical corticosteroids, or immunomodulatory drug use (59.09% vs. 60.61%, p = 0.66). The distribution of surgeries between the two surgeons was also similar post-matching (p = 0.73), further supporting balance in surgical technique and experience. The median follow-up time after surgery was 33.6 months (IQR: 18.1–58.1) and did not significantly differ between groups (32.08 months for hydrophilic vs. 34.97 months for hydrophobic IOLs, p = 0.07).

### Initial and preoperative visual acuity

The baseline CDVA was comparable between the hydrophilic and hydrophobic IOL groups both before and after propensity score matching. Prior to matching, the median initial CDVA was 1.0 LogMAR (IQR: 0.5–1.6) in the hydrophilic group and 0.8 LogMAR (IQR: 0.6–1.0) in the hydrophobic group (*p* = 0.79; SMD = 0.125). After matching, the median initial CDVA remained similar between groups—1.0 (IQR: 0.5–1.6) in the hydrophilic group versus 0.8 (IQR: 0.6–1.0) in the hydrophobic group (*p* = 0.87; SMD = 0.095).

Likewise, preoperative CDVA was not significantly different between groups. Before matching, the median preoperative CDVA was 1.0 LogMAR (IQR: 0.8–1.8) for both IOL groups (*p* = 0.31; SMD = 0.111). Post-matching, both groups again showed median preoperative CDVA values of 1.0 (IQR: 0.8–1.8 for hydrophilic, 1.0–1.8 for hydrophobic), with no statistically significant difference (*p* = 0.32; SMD = 0.100). These findings indicate a well-balanced visual function status at baseline between treatment arms.

Table 2 summarizes etiology of uveitis in this cohort. The most common etiology identified was idiopathic uveitis, accounting for 30.46% of cases (n = 53). This was followed by Vogt-Koyanagi-Harada (VKH) disease, observed in 22.41% of patients (n = 39), and HLA-B27-associated uveitis, which constituted 9.77% (n = 17).

**Table 2:** Etiology of uveitis.

Etiology	Total (N = 174)	Hydrophilic IOL (N = 99)	Hydrophobic IOL (N = 75)
Idiopathic	53 (30.46%)	27 (27.27%)	26 (34.67%)
Vogt-Koyanagi-Harada disease	39 (22.41%)	20 (20.20%)	19 (25.33%)
HLAB27	17 (9.77%)	10 (10.10%)	7 (9.33%)
Pars planitis	12 (6.90%)	10 (10.10%)	2 (2.67%)
Behcet’s disease	11 (6.32%)	9 (9.09%)	2 (2.67%)
Herpetic anterior uveitis	10 (5.75%)	7 (7.07%)	3 (4.0%)
Sarcoidosis	10 (5.75%)	5 (5.05%)	5 (6.67%)
Psoriatic anterior uveitis	7 (4.02%)	5 (5.05%)	2 (2.67%)
Tuberculosis	6 (3.44%)	3 (3.03%)	3 (4.0%)
Herpetic retinitis	3 (1.72%)	0 (0%)	3 (4.0%)
Posner-Schlossman syndrome	2 (1.15%)	2 (2.02%)	0 (0%)
White-Dot syndrome	2 (1.15%)	0 (0%)	2 (2.67%)
Fuch’s uveitis syndrome	1 (0.57%)	1 (1.01%)	0 (0%)
Serpiginous choroiditis	1 (0.57%)	0 (0%)	1 (1.33%)

Subgroup analysis based on IOL material showed that among the 99 patients with hydrophilic IOLs, the most prevalent causes were idiopathic (27.27%), VKH disease (20.20%), and HLA-B27-associated uveitis (10.10%). Similarly, in the hydrophobic IOL group (n = 75), idiopathic uveitis remained the most frequent etiology (34.67%), followed by VKH (25.33%) and HLA-B27 (9.33%).

### Primary outcomes

PCO was significantly more common in eyes implanted with hydrophilic IOL compared to hydrophobic lens. Before propensity score matching, PCO developed in 44.44% of the hydrophilic group versus 18.67% of the hydrophobic group (*p* < 0.001). This significant difference persisted after matching, with PCO present in 43.94% of hydrophilic IOL eyes compared to 19.70% of hydrophobic IOL eyes (*p* = 0.003), indicating a clear advantage in capsular biocompatibility with hydrophobic lenses. In the univariable Cox regression analysis, the use of hydrophobic intraocular lenses (IOLs) was significantly associated with a lower hazard of PCO compared to hydrophilic IOLs (HR = 0.33; 95% CI, 0.17–0.64; p = 0.0006). This association remained statistically significant in the multivariable model after adjusting for intraoperative use of iris retractors intraoperative steroid use and postoperative steroid use (adjusted HR = 0.35; 95% CI, 0.18–0.68; p < 0.05).

The median time from surgery to the first detection of PCO did not significantly differ between groups. After matching, the median time to PCO diagnosis was 8.73 months (IQR: 1.53–21.37) in the hydrophilic group and 17.40 months (IQR: 6.2–28.6) in the hydrophobic group (*p* = 0.13), suggesting a trend toward later onset in the hydrophobic IOL group, though not statistically significant.

Similarly, the need for Nd:YAG laser capsulotomy was significantly reduced in eyes receiving hydrophobic IOLs. In the matched cohort, 30.30% of hydrophilic IOL patients required the procedure compared to only 9.09% in the hydrophobic IOL group (p = 0.002). The univariable analysis demonstrated an HR of 0.20 (95% CI, 0.08–0.50; p = 0.0001), and this protective effect persisted in the adjusted multivariable model (adjusted HR = 0.23; 95% CI, 0.09–0.56; p < 0.05). In the matched cohort, the median CDVA prior to undergoing Nd:YAG laser capsulotomy was similar between groups. The median LogMAR VA in the hydrophilic IOL group was 0.7 (IQR: 0.5–1.0), while the hydrophobic group demonstrated a median VA of 0.6 (IQR: 0.5–0.6). Although there was a trend toward better visual acuity in the hydrophobic group prior to capsulotomy, this difference did not reach statistical significance (*p* = 0.21). These findings suggest that the indication for Nd:YAG laser treatment was based on similar thresholds of visual decline across both groups.

The median duration from surgery to Nd:YAG capsulotomy was slightly longer in the hydrophilic group (26.14 months; IQR: 10.33–40.75) compared to 19.55 months (IQR: 8.07–28.6) in the hydrophobic group, though this difference was not statistically significant (*p* = 0.58).

### Postoperative uveitis recurrence

There was no statistically significant difference in the rate of postoperative uveitis reactivation between the hydrophobic and hydrophilic IOL groups. The univariable analysis yielded an HR of 0.79 (95% CI, 0.35–1.76; p = 0.560), and the adjusted multivariable model showed a similar non-significant trend (adjusted HR = 0.83; 95% CI, 0.37–1.88; p = 0.643) (Table 3).

**Table 3:** Univariable and multivariable analyses of the association between two treatment groups (hydrophillic IOL versus hydrophobic IOL) and clinical outcomes.

Outcomes variables	Univariable analysis			Multivariable analysis^*^		
	HR	95%CI	P-value	aHR	95%CI	P-value
PCO occurrence	0.3339	0.17306-0.64412	0.0006	0.3522	0.1816-0.6834	<0.05
Nd:YAG capsulotomy	0.1999	0.0801-0.4986	0.0001	0.2254	0.0902-0.5630	<0.05
Postoperative uveitis reactivation	0.7887	0.3533-1.7606	0.560	0.8343	0.3699-1.8813	0.643

**Abbreviations:** IOL: Intraocular lens; PCO: Posterior capsule opacification; Nd:YAG: Neodymium-doped yttrium aluminum garnet

*Adjusted by intraoperative iris retractor/steroid used, and postoperative steroid used

### Postoperative visual outcomes

#### Final visual acuity and postoperative improvement.

The CDVA at the last follow-up visit and the improvement in visual acuity following cataract surgery were comparable between the hydrophilic and hydrophobic intraocular lens (IOL) groups. Prior to propensity score matching, the median logMAR CDVA at the final visit was 0.2 (IQR: 0.0–0.3) in both groups, with no statistically significant difference (*p* = 0.78; SMD = 0.139). After matching, the final visual acuity remained similar, with both groups again showing a median of 0.2 (IQR: 0.0–0.3) (*p* = 0.35; SMD = 0.078).

Visual acuity improvement, measured as the change in logMAR CDVA from preoperative to final follow-up, also showed no significant difference between groups. Before matching, the median LogMAR VA improvement was –0.8 (IQR: –1.6 to –0.6) in both groups (*p* = 0.55; SMD = 0.037). After matching, the hydrophilic group demonstrated a median improvement of –0.8 (IQR: –1.6 to –0.4), while the hydrophobic group improved by –1.0 (IQR: –1.6 to –0.7), a difference that remained statistically non-significant (*p* = 0.34; SMD = 0.134). These findings highlight that, in the absence of other ocular comorbidities and with well-controlled preoperative management, cataract surgery can result in favorable visual outcomes and improved visual acuity, irrespective of the intraocular lens (IOL) model used.

The incidence of postoperative posterior synechiae was also analyzed. After matching, 3.03% of patients in the hydrophilic group and 9.09% in the hydrophobic group developed new posterior synechiae postoperatively, a difference that was not statistically significant (p = 0.15; SMD = 0.254).

## Discussion

This study demonstrates that hydrophobic IOL provide superior capsular biocompatibility in patients with uveitis undergoing cataract surgery compared to hydrophilic IOL. Patients receiving hydrophobic IOLs experienced significantly lower rates of PCO and Nd:YAG capsulotomy, without a corresponding increase in the rate of postoperative uveitis reactivation.

PCO is the most common long-term complication following cataract surgery, and its development is influenced by IOL material and edge design [7]. IOL design, particularly sharp posterior optic edges create a capsular bend and reduced residual LECs migration, which is a primary factor in PCO development [7,9,10]. Hydrophobic IOLs have been consistently associated with lower rates of PCO in both uveitic and non-uveitic eyes [14,16,21]. Lower capsular biocompatibility of hydrophilic IOLs can be attributed to lower fibronectin adsorption and poorer capsule adhesion, which allows the migration and proliferation of LECs between the IOL and posterior capsule [14,22]. Our findings align with studies demonstrating that hydrophilic acrylic IOLs have greater susceptibility to PCO formation due to their higher water content and greater bioreactivity [14,23]. Differences in IOL design may influence postoperative outcomes. Indeed, the hydrophilic IOL used in our study (Bi-Flex 677AB) is a one-piece design, whereas the hydrophobic IOL (Sensar AR40e) is a three-piece model. It is recognized that the haptic-optic junction in some one-piece IOLs may allow lens epithelial cell migration, contributing to higher rates of PCO. Mian et al. [24] reported that three-piece IOLs were associated with significantly lower rates of posterior capsulotomy compared to one-piece lenses. However, Buehl et al. [9] found no significant difference in PCO or capsulotomy rates between the two designs.

The reduction in Nd:YAG capsulotomy rates observed with hydrophobic IOLs in our study is also consistent with prior reports [14,23,25]. Nd:YAG capsulotomy not only reflects clinically significant PCO but can itself lead to complications such as increased intraocular pressure and macular edema, which are particularly concerning in uveitic eyes [26].

Another important finding of our study is the low rate of postoperative uveitis reactivation observed in both groups. This underscores the importance of achieving quiescent inflammation before surgery and using appropriate perioperative immunosuppressive management. Our results reinforce that, under these conditions, cataract surgery can be performed safely in uveitic eyes without a high risk of disease reactivation.

The rate of postoperative uveitis reactivation was not significantly different between the hydrophilic and hydrophobic groups. This suggests that with appropriate perioperative immunosuppression and preoperative control of inflammation, both IOL materials can be safely implanted in uveitic eyes [13,17,27,28]. Interestingly, Roesel et al. reported no significant differences between hydrophilic and hydrophobic lenses in terms of PCO incidence or the need for Nd:YAG laser capsulotomy [28]. However, their study featured a relatively short follow-up period of only six months, which may have limited the detection of these long-term complications. In contrast, the present study had a considerably longer median follow-up duration of 33.6 months, during which the median time to Nd:YAG capsulotomy was 25.25 months (IQR: 10.4–38.63). This extended observation period may explain the discrepancy in findings and highlights the importance of long-term follow-up in evaluating postoperative outcomes in uveitic eyes.

Another notable finding from our study is the comparable improvement in corrected distance visual acuity (CDVA) between the hydrophilic and hydrophobic IOL groups, despite differences in PCO and Nd:YAG capsulotomy rates. This suggests that when preoperative inflammation is well-controlled and appropriate perioperative management is applied, favorable visual rehabilitation can be achieved irrespective of IOL type. These results emphasize that while hydrophobic IOLs offer long-term advantages in minimizing secondary interventions, such as laser capsulotomy, hydrophilic IOLs can still deliver satisfactory visual outcomes in selected cases. However, given the significantly lower rate of Nd:YAG capsulotomy in the hydrophobic group, the overall cost burden associated with laser treatment for PCO is likely to be reduced. Therefore, IOL selection should be individualized, taking into account patient-specific factors such as anatomical considerations, uveitis etiology, risk of postoperative complications, and the potential economic impact of secondary procedures.

The strengths of this study include the use of propensity score matching to control for confounding factors, a relatively long median follow-up of nearly three years, and standardized surgical techniques. However, the study’s limitations include its retrospective design, single-center setting with a limited number of patients, diverse underlying causes of uveitis that may influence inflammation activity and severity, and the use of specific IOL models, which may restrict the generalizability of the findings. Additionally, due to the retrospective design of the study, objective grading of posterior capsule opacification and detailed documentation of cataract severity or morphological types were not consistently available, limiting our ability to perform standardized assessments or subgroup analyses based on cataract characteristics.

In conclusion, hydrophobic IOLs provide superior outcomes in terms of PCO and Nd:YAG capsulotomy in uveitic eyes, without increasing inflammation recurrence. These findings support their use as the preferred IOL material in patients with controlled uveitis undergoing cataract surgery.
